# It is not just muscle mass: a review of muscle quality, composition and metabolism during ageing as determinants of muscle function and mobility in later life

**DOI:** 10.1186/2046-2395-3-9

**Published:** 2014-12-01

**Authors:** Robin A McGregor, David Cameron-Smith, Sally D Poppitt

**Affiliations:** School of Biological Sciences, University of Auckland, Auckland, New Zealand; Human Nutrition Unit, University of Auckland, Auckland, New Zealand; Liggins Institute, University of Auckland, Auckland, New Zealand; Department of Medicine, University of Auckland, Auckland, New Zealand; Riddet Institute, Palmerston North, New Zealand

**Keywords:** Muscle mass, Muscle composition, Muscle metabolism, Muscle function, Mobility, Ageing, Sarcopenia

## Abstract

Worldwide estimates predict 2 billion people will be aged over 65 years by 2050. A major current challenge is maintaining mobility and quality of life into old age. Impaired mobility is often a precursor of functional decline, disability and loss of independence. Sarcopenia which represents the age-related decline in muscle mass is a well-established factor associated with mobility limitations in older adults. However, there is now evidence that not only changes in muscle mass but other factors underpinning muscle quality including composition, metabolism, aerobic capacity, insulin resistance, fat infiltration, fibrosis and neural activation may also play a role in the decline in muscle function and impaired mobility associated with ageing. Importantly, changes in muscle quality may precede loss of muscle mass and therefore provide new opportunities for the assessment of muscle quality particularly in middle-aged adults who could benefit from interventions to improve muscle function. This review will discuss the accumulating evidence that in addition to muscle mass, factors underpinning muscle quality influence muscle function and mobility with age. Further development of tools to assess muscle quality in community settings is needed. Preventative diet, exercise or treatment interventions particularly in middle-aged adults at the low end of the spectrum of muscle function may help preserve mobility in later years and improve healthspan.

## Review

### Introduction

Maintaining mobility and health into old age is a major current challenge [1, 2]. The development of mobility limitations leads to reduced capacity for activities of daily living and impairs quality of life [3–5]. Impaired mobility is often a precursor of functional decline, disability and the development of frailty, which in turn leads to increased demand on primary caregivers and healthcare providers. Maintenance of mobility in middle age and old age is dependent on multiple components in muscles, bones, tendons, ligaments and joints and the impairment in any tissue can result in reduced mobility that in turn causes accelerated functional decline and disability. In older adults, mobility limitations have been defined as the self-reported inability to walk a mile, climb stairs or perform heavy housework [6]. During middle age, an acute injury such as a muscle tear can immediately limit mobility, but mobility and muscle function otherwise both vary over a wide spectrum. Lifelong physical exercise can preserve muscle structure and function in well-trained old-aged men comparable to that of active men four decades younger [7]. Even differences in mid-life leisure time physical activity can positively reduce the risk of impaired mobility in old age, although occupational physical activity in mid-life may have a negative impact on mobility in old age [8]. Determining ways to assess and identify middle-aged adults at the lower end of the muscle function spectrum compared to peers their own age would allow targeted interventions in mid-life to improve muscle function, which in turn may help preserve mobility in old age and hence increase healthspan.

Sarcopenia, originally described over two decades ago as the age-related loss of skeletal muscle mass [9], is a well-established factor associated with decreases in muscle strength and impaired mobility [10]. Loss of muscle mass appears to precede loss of bone during physical inactivity caused by mechanical unloading [11]. Cross-sectional studies in older adults and across the adult lifespan have shown that age is associated with a decline in muscle mass and muscle strength generally from the third or fourth decade onwards [12, 13]. But estimates of the rate of muscle loss per year vary between cross-sectional studies from 0.4%–2.6% per year [14]. Estimates of the rate of muscle strength loss during ageing are reported to be higher at 1%–3% per year, while the decline in muscle power is reported to be greater still [15]. Cross-sectional studies have primarily relied on indirect estimates of muscle mass such as dual X-ray absorptiometry (DXA) or bioelectrical impedance (BIA), which have been suggested by some to underestimate age-related changes in muscle mass [16]. Even though MRI or CT scans can provide an accurate measure of muscle cross-sectional area and also muscle composition [17, 18], neither are practical for assessment in community settings such as gyms, workplaces or health clinics.

Several longitudinal studies suggest that muscle mass alone cannot fully explain the loss of muscle strength and physical function in older adults [19–22]. Estimates of the rate of change in muscle strength with age derived from a cross-sectional cohort have also been suggested to underestimate actual yearly changes in muscle strength [19]. In the Health, Ageing, and Body Composition (Health ABC) study, the decline in muscle strength during ageing was reported to be two- to five fold greater than the loss of muscle mass in older adults aged 70–79 years over a 3-year follow-up period [20]. Furthermore, there was wide inter-individual variability in changes in muscle cross-sectional area and muscle strength in older adults, such that muscle mass and muscle strength were well-preserved in some individuals but not others [20]. In a small cohort of healthy men aged 65 years, thigh muscle cross-sectional area (CSA) decreased 1.2% per year, while thigh muscle strength decreased 1.3% per year during a 12-year follow-up period [23]. In a healthy Scandinavian cohort of older men and women aged 75 years, grip strength decreased 15%, while fat-free mass assessed by BIA decreased only 2.1% over 5 years [24]. This year, a large Japanese cohort study (*n* = 3952) including males and females aged 40–70 years reported trivial decreases in skeletal muscle mass measured by DXA over a 12-year follow-up period except in middle-aged men [25]. However, the authors acknowledge that the trivial changes in skeletal muscle mass observed may be partly due to participants being informed of their strength test results and training to improve their strength before subsequent follow-up tests [25]. Nevertheless, this highlights that lifestyle changes such as the adoption of resistance exercise in mid-life can help preserve muscle mass over the following decade. Taken together, evidence of a discordance between changes in muscle mass and muscle strength suggests that other factors related to muscle quality must make a contribution to age-related declines in muscle function and mobility [25–27]. While loss of muscle mass is a contributor to increased weakness in old age, the relationship between strength [28] and various aspects of force production with muscle size is far less robust in older, overweight or obese adults compared to younger adults [29, 30]. In the Health ABC study, 25% of males and 31% of females gained lean mass during the 3-year follow-up period, but this did not prevent increased weakness in older adults aged 70–79 years [19, 20]. This may be attributable to increases in BMI and adiposity. Greater gains in BMI over time are associated with higher odds of lower muscle quality based on the ratio of grip strength to arm lean mass [31]. Maintenance of muscle mass also did not prevent the loss of muscle strength observed in the Health ABC study, which highlights the importance of muscle quality as a contributor to muscle strength in older adults.

Muscle quality is closely intertwined with muscle strength, as muscle quality is typically defined as muscle strength or power per unit of muscle mass [32], although as yet, there is no universal consensus definition or assessment method for muscle quality. Given that both muscle strength and power are related to functional activities in older adults [33, 34], it could be argued that directly assessing muscle strength or power rather than muscle mass or quality would be more effective to identify middle-aged adults at higher risk of impaired mobility in later life. Home-based strength tests may be useful to assess changes over time in the same individual, but the difficulties with standardising a home-based test hampers evaluation against age-related strength norms. Grip strength is relatively straightforward for health workers or clinicians to assess providing equipment is available and age-related norms exist to stratify adults at risk of impaired mobility [35, 36]. Nevertheless, it would be advantageous to develop a muscle quality assessment tool for middle-aged adults in community settings that can provide information on muscle quality relative to age. A muscle quality assessment tool may then be used to identify individuals who would benefit from lifestyle interventions to improve muscle quality. Multiple factors have the potential to influence muscle quality including muscle composition (e.g. architecture, fibre type), metabolism, fat infiltration, fibrosis and neural activation. In the following, we discuss factors underpinning muscle quality and potential ways to assess muscle quality.

#### Muscle size, fibre type and contractile characteristics

Muscle size is determined by muscle fibre type and number. Muscle CSA is positively associated with muscle strength in young lean individuals [37]. Smaller, weaker muscles are typically observed in elderly compared to younger individuals. However, muscle cross-sectional area and lower limb skeletal muscle volume is also associated with greater adiposity in males and females (BMI 19-36) [38], which may be due to the presence of intermuscular lipid or non-contractile components. Muscle fibre types can be broadly categorised based on metabolic characteristics, such that type IIb fibres predominantly generate energy via non-oxidative pathways for rapid high force production, type I fibres predominantly generate energy via oxidative pathways for prolonged low force production whereas type IIa fibres are capable of generating energy via oxidative and non-oxidative pathways [39]. Type I fibres have a relatively small CSA compared to type IIb fibres, but their oxidative capacity is significantly greater [40]. Some observations of muscle fibres in advanced age indicate that there may be a shift in muscle fibre composition, with a progressive loss of type IIb fibres [41], but others show no association between fibre composition and age [42]. Type IIb muscle fibres are recruited during high-intensity activity, whereas type I muscle fibres are recruited during low-intensity activities of daily living such as walking. The loss of either type of muscle fibre with age may be attributable to changing activity levels, leading to disuse and denervation. There are no studies of the longitudinal changes in muscle fibres in middle-aged humans. However, a longitudinal study in middle-aged rheus monkeys reported that a decrease in muscle fibre CSA was a significant contributor to the loss of muscle mass in the vastus lateralis with age [43]. A follow-up study identified that a shift in metabolism occurred in advance of the onset of sarcopenia in middle-aged rhesus monkeys and fibre-type distribution shifts towards myosin heavy chain (MHC) type II fibres and mitochondrial oxidative phosphorylation was significantly reduced [44]. In a healthy ageing rodent model, muscle fibre CSA was observed to decline from 24 months, whereas muscle mass and fibre number were decreased after 30 months [45]. The relatively late decline in muscle CSA and mass may be attributable to the consistent diet and activity levels of lab-raised rodents. The limited evidence available in elderly individuals suggests there is discordance between changes in muscle fibre type, muscle CSA and strength [23]. At the single fibre level, contractile properties of type I and type II muscle fibres appear to be preserved independent of mobility limitation [46] although there are conflicting reports. D’Antona et al. [47] suggested that maximal shortening velocity is lower in single fibres from elderly adults due to a shift in MHC isoform distribution to a more hybrid pattern [47]. Larsson et al. [48] observed a lower maximal shortening velocity in MHC I and IIa fibre types in elderly and very active elderly compared to that in young adults [48]. In contrast, Trappe et al. observed no age-related differences in single fibre contractile velocity of MHC I and IIa fibres [49]. Studies of single fibre contractile characteristics typically involve very few subjects and in earlier studies physical activity may have been a confounding factor, which can influence contractile characteristics independent of age [49]. If single muscle fibre contractile properties are indeed preserved with age, this would suggest that differences in skeletal muscle function are related to quantitative changes in muscle fibre size or number rather than qualitative changes in contractile properties.

### Muscle architecture

In addition to observations of possible age-related fibre type changes, muscle architecture including fascicle length and pennation may change with age. Muscle architecture is the arrangement of fibres within the muscle either in a parallel or pennation pattern, which determines fascicle length (*L*_f_), pennation angle (*θ*) and CSA. Ultrasonography assessment of *gastrocnemius medialis* fascicle length and pennation angle were reported to be smaller in elderly adults aged 70–81 years compared to younger adults aged 27–42 years [50]. Accelerated ageing models, such as several weeks bed rest, have not been reported to cause any alteration in the pennation angle of *vastus lateralis* muscle [51]. However, modest improvements in muscle architecture are possible with 4–5 weeks of resistance training, and notably changes in muscle architecture appear to precede changes in muscle size in young healthy adults [52]. Therefore, non-invasive techniques such as ultrasound have potential to identify middle-aged or older adults who would benefit from interventions to improve muscle architecture. To be of practical utility, an ultrasound device ideally needs to ensure a fixed limb position and probe position to limit measurement error.

#### Muscle aerobic capacity

The metabolic characteristics of muscle are important determinants of muscle quality and, in turn, muscle function in both middle-aged and older adults. There is evidence that aerobic capacity, which is the maximal ability to use oxygen to meet the energy demands of physical activity, may decline at an accelerated rate after the age of 50 years [53]. Aerobic capacity is a strong predictor of mobility assessed by gait speed in older adults [54]. Aerobic capacity reflects not only cardiovascular adaption to transport oxygen but also adaptations within muscle to use oxygen to meet the energy demands of physical activity. Cross-sectional evidence from healthy men and women aged 18–90 years indicates that mitochondrial DNA, mRNA abundance and energy (ATP) production all decrease with age [55]. Skeletal muscle mitochondrial capacity and efficiency, as well as whole body peak aerobic capacity have been shown to be associated with gait speed [56]. Furthermore, individuals who are aerobically active in young, mid- and later life have higher muscle strength than their sedentary peers of the same age [57]. Regular physical activity is a powerful stimulus to help retain or improve aerobic capacity in middle-aged adults, which in turn can help maintain muscle function and mobility into old age [57].

The assessment of maximal aerobic capacity (VO_2_max) in middle-aged adults would provide an important functional indicator of muscle quality beyond muscle mass. While direct methods of VO_2_max assessment require gas analysis equipment, indirect low-cost methods can provide an estimate of VO_2_max based on extrapolation from heart rate, work performed in a set amount of time or time to complete a set amount of work.

Skeletal muscle is responsible for a large proportion of whole-body glucose uptake, therefore age-related changes in muscle mass and composition can lead to increased insulin resistance and hence reduced capacity for insulin-mediated glucose disposal. In a cross-sectional cohort of healthy non-diabetics aged 65 years, relative muscle mass was inversely associated with glucose tolerance and insulin resistance [58]. Evidence of the link between muscle strength and insulin resistance is more equivocal. Muscle strength adjusted for body mass index (BMI) was reported to be negatively associated with insulin resistance in a large population-based study (*n* = 968) of older women but not men after adjustment for confounders [59]. On the other hand, analysis of the US National Health and Nutrition Examination Survey (NHANES) revealed no association between muscle leg strength and insulin resistance in men or women aged over 50 years [60]. Gait speed tests have been reported in some studies to be inversely associated with insulin resistance. These findings suggest that insulin resistance may provide an indicator of poor muscle quality underpinning low levels of physical fitness and poor scores on gait speed tests. Both regular aerobic and resistance type exercise over 6 months have been shown to improve glucose disposal and skeletal muscle metabolism in older overweight or obese men aged approximately 63 years [61]. Insulin resistance is also known to be closely associated with intermuscular fat [62].

#### Intermuscular adipose tissue

Over the past decade, intermuscular adipose tissue (IMAT) has emerged as an important factor underpinning muscle quality and also may be a predictor of muscle function in older adults [27]. IMAT is an ectopic adipose tissue depot located under the fascia and within the muscle. IMAT encompasses a variety of interchangeable terms including intramuscular fat and low-density lean tissue which describes fat stored between muscle fibres and intermuscular fat which describes fat located underneath the fascia [63]. IMAT can be assessed indirectly using muscle attenuation calculated from a computed tomography (CT) or magnetic resonance imaging (MRI) scan which closely correlates with direct measurements of muscle lipid content [18]. Individuals with similar thigh circumference may have distinctly different muscle function because of the proportion of IMAT to contractile elements. In older adults with a variety of comorbidities, IMAT assessed by MRI was reported as the strongest predictor of mobility in older individuals, although strength and quadriceps lean tissue explained some of the variance in mobility in this study [64]. Lipid storage and infiltration into muscle may also be a good marker of metabolic profile. For example, in young- to middle-aged adults (24–48 years) with a range of body composition from lean to obese, IMAT assessed by CT was reported to be significantly associated with insulin resistance assessed by glucose clamp [62]. Another study reported IMAT to be positively correlated with higher fasting plasma glucose and lower glucose tolerance in older adults [65].

IMAT appears to be reversible with physical activity. One report showed that 6 months of aerobic exercise training and weight loss decreased IMAT of the leg, while fasting plasma glucose and glucose tolerance were concomitantly improved in men aged 60 years [65]. Conversely, in healthy young adults, 4 weeks of an enforced decrease in physical activity induced by unilateral lower limb suspension led to a 15%–20% increase in IMAT in the thigh and calf, respectively [66]. Strength loss was reported to be associated with the increase in IMAT, after adjustment for loss of muscle mass and initial values at baseline [66]. Progressive IMAT accumulation in middle-aged or older adults may lead onto fibrosis and further impair muscle function and mobility.

It would be advantageous to develop ways to assess muscle quality based on IMAT using methods besides CT or MRI that can be used in a community setting. Echo intensity calculated from an ultrasound scan appears to inversely correlated with radiological density calculated from a CT scan, therefore may be used as a surrogate measure of IMAT and non-contractile elements [67]. In active elderly women, echo intensity was reported to be negatively correlated with functional performance measures including sit-to-stand time and usual gait speed [68]. Furthermore, in healthy women aged 51–87 years, echo intensity was reported to be significantly correlated with quadriceps strength after adjustment for age and muscle thickness [69]. While portable ultrasonography machines are available, they still require a skilled sonographer to perform the scan and interpret the results. In addition, echo intensity is not directly comparable between studies due to differences in ultrasonography machines. An alternative technology is electrical impedance myography (EIM) that can measure multi-frequency electric impedance; healthy muscle shows different reactance and phase dependence at different frequencies. EIM was reported to be a potential biomarker of neurodegenerative disorder amyotrophic lateral sclerosis (ALS) [70]. Interestingly, in mobility-limited elders aged 78 years, EIM was reported to be correlated with CT-determined muscle attenuation and strength [71]. In addition, in a large cohort of individuals aged 19–50 years and 60–85 years, a small handheld EMI device was used to assess several lower and upper extremity muscles [72]. Lower reactance and resistance values were measured in lower extremity muscles, but not upper extremity muscles, of the older compared to the younger adults [72]. These differences were more pronounced in men than women [72]. It will be important in future to test the reliability and validity of EIM for measuring and monitoring changes in muscle quality over time in response to lifestyle interventions including exercise or diet.

#### Muscle fibrosis

Muscle fibrosis can occur due to the impairment in the muscle repair process after an injury. The process of fibrosis involves the deposition of collagen and extracellular matrix (ECM) proteins instead of proteins necessary to repair and restore tissue function [73]. Fibrosis is also observed in different tissues due to excess fat accumulation [74, 75]. The development of pathological fibrosis in tissue is the end result of a series of events including injury, infiltration of inflammatory cells, tissue degeneration and proliferation of fibroblasts which result in remodelling of tissue architecture [73]. There is no direct evidence of muscle fibrosis with age in humans due to the difficultly in assessing fibrosis in population studies. Evidence from microarray studies indicates that fibrosis-related transcripts are differentially expressed in older (65–80 years) compared to younger adults (19–29 years) [76]. Evidence from aged mice indicates that muscle stem cells tend to convert from a myogenic to a fibrogenic lineage [77]. Furthermore, in elderly humans, muscle stem cell populations are reported to be lower than their younger counterparts [78]. Currently, the presence of fibrosis in the skeletal muscle of older adults is speculative. However, this does not preclude that molecular changes governing cell structure, remodelling, collagen formation and fibrosis occur which have not been well studied. Tissue fibrosis is detectable using MRI, CT, or ultrasound imaging, but is used primarily for the detection of pathological conditions such as hepatic fibrosis. The contribution of fibrosis as a factor underpinning muscle quality requires further investigation.

#### Motor units and neuromuscular activation

Components of the neuromuscular system and neuromuscular activation are other potential factors underpinning muscle quality in middle and old age. Skeletal muscle fibres are organised in bundles of motor units; each motor unit is innervated by a motor nerve which connects to an alpha motoneuron in the spinal cord. Motor units undergo remodelling, denervation and re-innervation throughout the lifespan. Motor unit number estimated based on surface and intramuscular electromyographic signals during isometric contractions shows that motor units are reduced in the tibialis anterior muscle of men aged 65 years and further in men aged over 80 years compared to that in young men aged 25 years [79]. However, the reduced motor unit number was only related to strength in the men aged over 80 years [79]. Early loss of motor units due to death of a motor neuron or axonal degeneration may not lead to loss of strength due to successful remodelling and rennervation by an adjacent motor neuron. Impairments in neuromuscular activation influence the rate of force development and muscle power necessary for dynamic movements. Improvements in neuromuscular activation precede increases in muscle mass in response to resistance training; therefore, neuromuscular activation has been proposed as another measure of muscle quality [80]. Surface electromyography (EMG) can be used to assess neuromuscular activity; a recent study in older adults reported that neuromuscular activity and acceleration was impaired during dynamic leg extensions in mobility-limited compared to mobile older adults [81]. There was a lag between EMG detection and movement, as well as the rate and magnitude of the EMG signal in the mobility-limited older adults. The rate of neuromuscular activation was significantly associated with physical function scores [81]. Among the middle-aged and older adults tested without mobility limitations, there were no significant differences in EMG measures of neuromuscular activation [81]. Therefore, whether neuromuscular impairment precedes the development of mobility limitations is still equivocal. The surface EMG signal is sensitive to adipose tissue; therefore, changes in adipose tissue due to weight loss or exercise could hamper interpretation of the EMG signal, although in a research setting, this could be overcome by using intramuscular EMG instead of surface EMG.

### Way forwards

It will be important in future to better understand the main factors which underpin changes in muscle quality with age, which may well precede changes in muscle mass or be of greater functional significance in ageing muscles, with declining size. In addition, a universal consensus definition of muscle quality is necessary. Muscle quality is typically used to describe muscle strength or power per unit of muscle mass, therefore does not encompass muscle aerobic capacity which is closely associated with mobility and important for activities of daily living. The majority of studies on muscle quality to date focus on older adults aged 65 years. Currently, there is a large gap in our knowledge on the primary determinants of muscle quality in middle-aged adults, which significantly inhibits the opportunity to intervene appropriately with either dietary- and/or activity-focused programmes. The development of muscle quality assessment tools that encompass muscle quality and which are sensitive to small changes within muscle that precede a decline in muscle function would enable individuals to take preventative steps to maintain healthy muscle. Non-invasive imaging of muscle by MRI, CT and ultrasound can capture multiple factors related to muscle quality such as size, composition, intramuscular lipid and fibrosis in a research setting. However, new ways to assess muscle quality are needed that are practical in a community setting. Given that age-related changes in skeletal muscle occur slowly, large prospective trials of interventions to improve muscle quality will be important to provide evidence for recommendations to promote healthy ageing.

## Conclusions

Muscle quality is increasingly being recognised as an important determinant of muscle function. Muscle size, fibre type, architecture, aerobic capacity, intermuscular adipose tissue, fibrosis and neuromuscular activation all potentially contribute to muscle quality. Development of muscle quality assessment tools, which can be used in a community setting particular in middle-aged adults is a priority. It will be important that new tools are evaluated for reliability and validity. Finally, lifestyle interventions targeting middle-aged adults at the low end of the spectrum of muscle function hold the potential to help preserve mobility in old age and improve healthspan.
